# Anticancer Potential of Whey Proteins—A Systematic Review of Bioactivity and Functional Mechanisms

**DOI:** 10.3390/ijms262110406

**Published:** 2025-10-26

**Authors:** Selin Elmas, Meliha Fındık, Ramazan Kıyak, Gökhan Taşkın, Daniela Cîrțînă, Rodica Dîrnu, Natalia Guță, Roxana-Maria Mecu, Monica-Delia Bîcă

**Affiliations:** 1 Department of Food Engineering, Faculty of Agriculture, Uludag University, Bursa 16059, Turkey; 2 Department of Emergency Medicine, Faculty of Medicine, Balıkesir Üniversity, Balıkesir 10010, Turkey; 3 Faculty of Medical and Behavioral Sciences, “Constantin Brancusi” University of Târgu-Jiu, 210135 Targu Jiu, Romania

**Keywords:** whey proteins, anticancer effects, bioactive peptides, molecular mechanisms

## Abstract

Cancer remains a primary global health concern, with treatment-related side effects and malnutrition posing significant challenges to patient care and recovery. In recent years, there has been growing interest in the therapeutic potential of functional food components, especially whey proteins (WPs), due to their notable antioxidant, immunomodulatory, and anticancer properties. This systematic review explores the effects of WPs across various cancer types and assesses their value as supportive nutritional agents. A thorough literature search was conducted in PubMed, Scopus, and Web of Science databases, identifying 24 relevant studies published between 2000 and 2024. The selection process followed PRISMA guidelines. The evidence, drawn from both laboratory and clinical research, suggests that WPs may exert anticancer effects by inhibiting tumor cell growth, promoting apoptosis, enhancing antioxidant defenses, modulating immune activity, and influencing signaling pathways such as the PI3K/Akt, mTOR, and Wnt/β-catenin pathways. Colorectal, breast, and liver cancers emerged as the most extensively studied types. Additionally, the form of WP used—whether concentrate, isolate, or hydrolysate—appeared to influence both biological activity and clinical outcomes. Clinical findings suggest that WP supplementation may support nutritional status, mitigate the adverse effects of chemotherapy, and enhance the quality of life in cancer patients. While the preclinical data are compelling, further high-quality randomized controlled trials are needed to confirm these benefits and determine optimal use in clinical practice. This review highlights WPs as promising, well-tolerated nutritional agents with potential to enhance current cancer care strategies.

## 1. Introduction

Cancer remains one of the most pressing global health concerns, exerting a substantial burden on individuals, healthcare systems, and national economies. In 2020, there were approximately 19.3 million new cancer cases and nearly 10 million deaths worldwide [1]. Projections suggest that the number of new cases could reach 30 million annually by 2040, according to the International Agency for Research on Cancer [IARC], 2024. Population-based cancer registries (PBCRs) are essential for tracking trends in incidence, mortality, and survival across diverse populations, and their continued development is vital for informed public health planning [2]. Globally, breast, lung, colorectal, prostate, and stomach cancers account for over half of all diagnoses [1], highlighting the need for integrative strategies that support both disease management and patient well-being.

Despite progress in diagnostics and treatment, cancer incidence is steadily increasing. This rise is accompanied by therapy-related complications, including malnutrition and cachexia, which are particularly prevalent in patients receiving chemotherapy or radiotherapy. These complications often result in reduced physical capacity, impaired treatment adherence, and a lower quality of life [3]. Cancer cachexia—a multifactorial syndrome characterized by muscle wasting, weight loss, and metabolic disruption—affects up to 60% of patients and is responsible for nearly 20% of cancer-related deaths. Malnutrition, driven by tumor burden, inflammation, and treatment toxicity, has been reported in up to 85% of patients with advanced-stage disease and is associated with prolonged hospital stays, poorer outcomes, and increased mortality [4]. These challenges underscore the need for supportive approaches that extend beyond tumor targeting and address patients’ nutritional and functional needs throughout treatment.

Malnutrition remains one of the most common and underappreciated conditions in oncology care, affecting 40–80% of hospitalized patients [5]. It contributes to sarcopenia, immune dysfunction, decreased treatment tolerance, and more extended hospital stays, exacerbating the overall disease burden. Consequently, nutritional interventions have become a central element of supportive cancer care. Functional food ingredients with immunonutritional and cytoprotective properties are being increasingly explored as adjunctive strategies to mitigate adverse effects and enhance therapeutic outcomes [6].

Among these, whey proteins (WPs) have emerged as an up-and-coming class of bioactive compounds. Constituting around 20% of bovine milk protein, WPs are composed of several peptides—such as lactoferrin, α-lactalbumin, and β-lactoglobulin—known for their antioxidant, immunomodulatory, and anticancer effects [5,6]. Preclinical research has demonstrated that WP can reduce oxidative stress in the tumor microenvironment, induce apoptosis (via caspase-3/7 activation), and arrest the cell cycle in the G0/G1 phase [7]. A high cysteine content enhances intracellular glutathione synthesis, thereby protecting against chemotherapy-induced toxicity. WP has also been shown to inhibit oncogenic pathways such as IGF-1 and PI3K/AKT/mTOR [8].

WPs are available in three main formulations: concentrate (WPC), isolate (WPI), and hydrolysate (WPH), each differing in protein content, peptide composition, and digestibility. WPC (35–80% protein) retains more biologically active peptides, WPI (>90% protein) is highly purified with minimal fat and lactose, and WPH contains short-chain peptides derived from enzymatic hydrolysis, offering enhanced digestibility and lower allergenicity [5,9,10]. These formulation-specific characteristics influence bioavailability and therapeutic potential, necessitating a comparative evaluation of their effects.

Recent clinical trials have reinforced the therapeutic relevance of WPs in cancer care. Supplementation has been linked to improved body composition, increased muscle strength, and reduced complications such as neutropenia and oxidative stress, particularly in patients with colorectal, breast, and liver cancers undergoing chemotherapy [11,12,13]. These benefits are primarily attributed to WPs’ unique amino acid profile and bioactive properties, which support immune function and metabolic resilience.

While several systematic reviews have explored the anticancer properties of WPs, most have focused on a single cancer type or formulation and often rely heavily on preclinical studies. As a result, the available clinical evidence remains fragmented and lacks integration with molecular insights [7,8,14]. To date, no comprehensive review has systematically compared the effects of different WP formulations across multiple cancer types.

This review aims to fill that gap by synthesizing current knowledge on the molecular mechanisms and clinical outcomes associated with WP use in cancer patients (Table 1). It evaluates the impact of WPC, WPI, and WPH in diverse malignancies and offers formulation-specific insights to guide their clinical application as supportive agents in oncology.

Whey Proteins and Colorectal Cancer.

Colorectal cancer (CRC) remains one of the leading causes of cancer-related mortality worldwide [1]. In recent years, increasing attention has been directed toward supportive nutritional strategies, including whey protein (WP), which is rich in bioactive components such as lactoferrin, β-lactoglobulin, and α-lactalbumin, each exhibiting potent antioxidant, immunomodulatory, and anti-inflammatory properties [15,16,17].

In a randomized, placebo-controlled trial, Mazzuca et al. (2019) showed that daily supplementation with a purified WP formulation improved skeletal muscle mass and significantly reduced chemotherapy-related hematologic and gastrointestinal toxicities in CRC patients [12]. Supporting this, Rabie et al. (2024) reported that WP supplementation enhanced nutritional status, immune function, and treatment tolerance in malnourished cancer patients across multiple clinical settings [17].

From a mechanistic perspective, WP-derived peptides have been shown to regulate gut microbiota composition, bolster endogenous antioxidant defenses, and suppress key oncogenic signaling cascades, including the Wnt/β-catenin and PI3K/AKT pathways [16]. Proteomic and preclinical data further reveal WP’s capacity to induce apoptosis, inhibit epithelial–mesenchymal transition (EMT), and limit metastatic progression in colorectal tumor models [8].

In conclusion, accumulating evidence suggests that WP exerts multifaceted benefits in the prevention and management of CRC through nutritional support, immune modulation, and direct anticancer mechanisms.

Whey Proteins and Breast Cancer.

Breast cancer remains the most commonly diagnosed malignancy among women worldwide and constitutes a significant cause of cancer-related morbidity and mortality. Whey acidic protein (WAP), a prominent component of whey, has been shown to suppress proliferation and invasion of human breast cancer cells (MCF-7 and MDA-MB-453) by inhibiting laminin degradation and downregulating angiopoietin-2 expression, indicating its potential as a protease-inhibitory therapeutic agent against tumorigenesis [18].

In a recent in vitro study, Sahna et al. (2023) evaluated the antitumor potential of goat milk-derived bioactive peptides on MCF-7 breast cancer cells. The trypsin-treated whey protein fractions significantly reduced cell viability and altered the expression of key oncogenic regulators, including pyruvate kinase M2 and mucin-1C, indicating that these peptides may act through diverse molecular pathways to exert cytotoxic effects [19].

Cheng et al. (2017) demonstrated that whey protein concentrate (WPC) enhanced the sensitivity of MDA-MB-231 triple-negative breast cancer cells to rapamycin by modulating intracellular redox homeostasis and activating the GSK3β/mTOR signaling cascade. This synergistic effect underscores the potential of WPC as an adjuvant strategy for overcoming therapeutic resistance in aggressive breast cancer phenotypes [20].

Singh et al. (2023) developed genipin-crosslinked whey protein nanoparticles loaded with doxorubicin and demonstrated that NAC-modified formulations (CyWD) significantly enhanced antitumor efficacy in triple-negative breast cancer (TNBC) models, suggesting whey protein-based nanocarriers as promising targeted delivery systems [21].

In addition to its anticancer and immunomodulatory effects, whey protein isolate (WPI) may also support psychological well-being in cancer patients. Xia et al. (2023) demonstrated that WPI alleviated tumor-induced depression-like behavior in 4T1 breast cancer-bearing mice by modulating hippocampal tryptophan metabolism and gut microbiota composition, suggesting WPI’s potential in mitigating cancer-related affective disorders [21,22].

Sabancılar and Durak (2022) reported that whey protein (WP) derived from cow’s milk exhibited marked dose- and time-dependent cytotoxic effects on MCF-7 breast cancer cells. The study also indicated that WP stimulated the production of key cytokines, including IL-2, IL-6, and TNF-α, suggesting a potential immunomodulatory mechanism underlying its antiproliferative activity [23]. Animal studies have also shown the protective effects of whey protein hydrolysate against mammary tumors [24].

Recent evidence underscores the multifaceted anticancer potential of whey proteins through various molecular mechanisms. Ramani et al. (2023) emphasized that whey protein and its components, particularly lactoferrin-derived peptides, can induce apoptosis via caspase activation and exhibit strong antioxidant effects due to their iron-binding capacity. These mechanisms not only contribute to reducing cancer risk but also provide therapeutic support by attenuating muscle atrophy and anorexia in cancer cachexia [25].

Whey Proteins and Other Cancer Types.

Beyond colorectal and breast cancers, whey proteins (WP) have demonstrated promising anticancer effects in various other malignancies. In hepatocellular carcinoma, WP concentrate significantly attenuated oxidative liver damage and tumor progression, primarily by enhancing hepatic antioxidant capacity and modulating lipid peroxidation [26]. Similar protective mechanisms have been observed in prostate cancer, where Rosa et al. (2020) reported that a fermented WP-based beverage exerted antiproliferative and pro-apoptotic effects on prostate cancer cells through caspase activation and mitochondrial disruption [27]. In hematologic cancers, Badr et al. (2021) found that camel milk-derived WP induced apoptosis in multiple myeloma cells via both intrinsic and extrinsic pathways, highlighting WP’s immunomodulatory and cytotoxic potential [28]. Additionally, the cytotoxic effects of WP have been extended to lung cancer models, where Ali and Elsebaie (2018) demonstrated WP’s capacity to reduce cell viability and enhance apoptotic responses in vitro [29]. Tsai et al. (2000) further showed that WP isolates augmented chemotherapeutic cytotoxicity in hepatoma cell lines, suggesting a synergistic role in combination therapies [30]. Collectively, these findings suggest that WP exerts multifaceted anticancer activities, ranging from redox regulation and apoptosis induction to enhanced drug sensitivity, across various cancer types, thereby supporting its broader application in integrative oncology.

## 2. Materials and Methods

This review was conducted in accordance with the PRISMA (Preferred Reporting Items for Systematic Reviews and Meta-Analyses) guidelines. A systematic literature search was conducted in the PubMed, Web of Science, and Scopus databases, spanning the period from 2000 to 2024. Various combinations of the terms “whey protein,” “cancer,” and “anticarcinogenic” were applied.

Only peer-reviewed articles with full-text availability were considered. Eligible studies included in vitro, in vivo, and human clinical trials directly investigating the effects of whey proteins or their bioactive fractions on cancer cells or cancer patients. Studies focusing on general dairy consumption, other protein sources, or non-cancer health effects of whey proteins were excluded.

To ensure methodological rigor, Embase and CENTRAL were not searched because of their substantial overlap with the selected databases and limited additional coverage relevant to the topic. Gray literature sources such as theses, conference abstracts, preprints, or institutional reports were excluded to maintain consistency and to include only studies that underwent formal peer review. This approach aligns with PRISMA 2020 recommendations for transparent reporting of search scope and inclusion criteria.

Two reviewers independently assessed the methodological quality and risk of bias of the included studies. In vitro and in vivo studies were evaluated according to reproducibility criteria (cell line characterization, exposure conditions, controls, and reporting completeness). At the same time, clinical trials were appraised using the Cochrane Risk of Bias 2.0 tool. Each domain was classified as low risk, some concerns, or high risk of bias. Discrepancies were resolved by consensus. Additionally, the certainty of the overall evidence for clinical outcomes was assessed using the GRADE approach.

**Figure 1:**
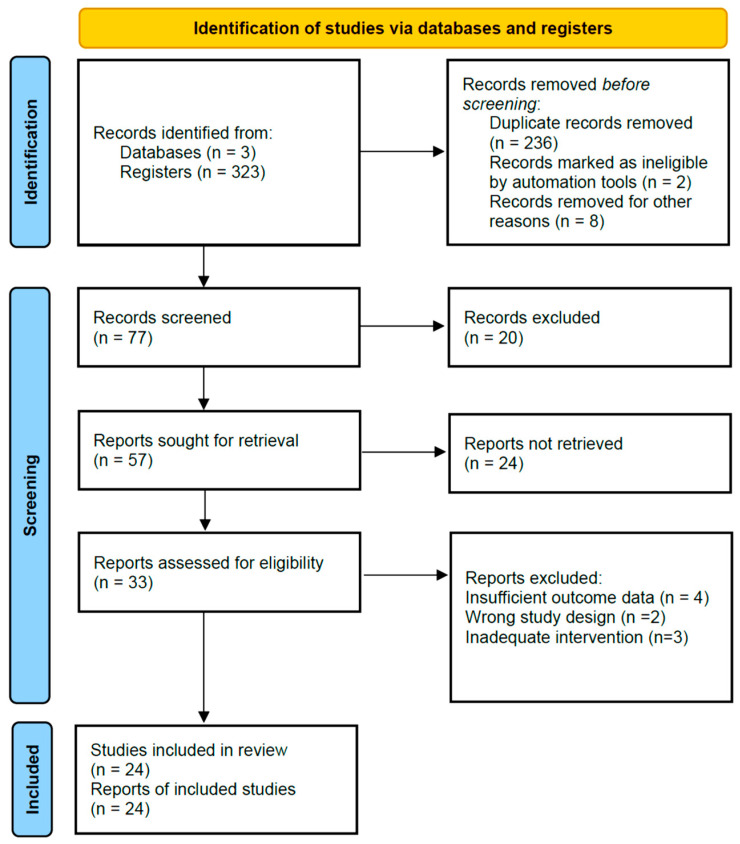
PRISMA 2020 Flowchart of the study selection process.

From an initial pool of 323 articles, 77 were selected for full-text review after title and abstract screening. Following full-text evaluation according to inclusion and exclusion criteria, 24 studies were retained for analysis. Two independent researchers conducted the selection process (Figure 1).

Registration and Protocol.

This systematic review was not preregistered in any database. However, the review process strictly adhered to the PRISMA 2020 guidelines to ensure transparency, reproducibility, and methodological accuracy.

For each study, the following data were extracted using a standardized form: author(s) and year of publication, study design (in vitro, in vivo, clinical), type of whey protein investigated (isolate, hydrolysate, purified fraction, etc.), cancer type studied, primary biological mechanisms identified (e.g., apoptosis induction, antioxidant defense, immune modulation), primary outcomes, and key findings. A qualitative synthesis was then performed to integrate and summarize the evidence.

## 3. Results and Discussion

Whey protein is a functional by-product generated during cheese production, with an annual global production volume of 180–200 million tons, posing a significant environmental burden [31]. However, due to its high content of bioactive proteins (β-lactoglobulin, α-lactalbumin) and lactose, it can be converted into value-added products through methods such as membrane technologies, enzymatic hydrolysis, and crystallization [32].

Sweet whey is produced during the making of semi-hard and hard cheeses at a pH of 6.5, using rennet. In contrast, acidic whey is derived from products such as cream cheese, ricotta, paneer, and Greek yogurt, where casein is precipitated at a pH of approximately 4.6. Whey typically contains over 70% lactose and 7.5% to 14% protein, primarily composed of β-lactoglobulin, α-lactalbumin, and glycomacropeptide, the last of which is unique to rennet-treated whey. It also contains immunoglobulins, bovine serum albumin, lactoferrin, lactoperoxidase, and various minor proteins and peptides [9,33].

Whey protein concentrate (WPC), isolate (WPI), and hydrolysate (WPH) differ substantially in composition and bioactivity. WPC is typically produced using membrane-based techniques such as ultrafiltration or microfiltration and contains 35–80% protein. WPI, obtained through ion exchange chromatography or diafiltration, contains over 90% protein with minimal lactose and lipids, whereas WPH results from enzymatic hydrolysis that breaks protein chains into smaller peptides, enhancing digestibility and antioxidant capacity [9,10].

These protein fractions exhibit distinct differences in terms of industrial applications. For example, WPC is widely used in the food industry (e.g., dairy products, baking) due to its high emulsification and gel-forming capacity. At the same time, WPI is preferred for sports supplements and clinical nutrition products due to its high purity level. WPH, on the other hand, is used in infant formula and medical nutraceuticals due to its reduced allergenic potential and rapid amino acid absorption, thanks to its hydrolyzed form [5].

Whey components, particularly whey proteins (WPs), bioactive peptides (e.g., β-lactoglobulin, α-lactalbumin, lactoferrin), and their antioxidant properties have been extensively studied for their antitumor, antiproliferative, and antiangiogenic mechanisms in cancer prevention and treatment [14]. Whey proteins exhibit multiple mechanisms, including neutralizing reactive oxygen species (ROS) [16], inducing apoptosis through caspase-3 activation [34] and arresting the cell cycle in the G1/S phase via cyclin D1 inhibition [35], and exerting cytotoxic and antiproliferative effects in colorectal adenocarcinoma cells [36].

Among the 24 included studies, 15 were in vitro, seven were in vivo, and two were randomized controlled clinical trials. The most commonly studied cancer types were colorectal and breast cancers. Various whey protein formulations were investigated, with WPC being the most frequent. A comprehensive summary of study characteristics, along with risk of bias assessment, is provided in Table 2. Most clinical trials were assessed as having a low to moderate risk of bias, primarily due to small sample sizes and limited follow-up, whereas in vitro studies showed a higher risk due to incomplete reporting of replicates and controls. Animal studies were generally rated as low to some concerns.

The diversity of study designs, cancer types, and whey protein formulations indicates a broad interest in the potential cancer-preventive properties of whey protein in various contexts. However, this heterogeneity also limits cross-comparability and the ability to draw general conclusions regarding the efficacy of whey proteins in all cancer types or treatment settings.

The most commonly reported results were the induction of apoptosis, decreased cell viability, and a reduction in tumor incidence. Other common results included cytotoxic effects, improved nutritional status, and antioxidant effects. Multiple results were reported in many studies. For instance, Dreanca et al. (2022) demonstrated that whey-based formulations reduced tumor volume in colon carcinoma-bearing mice [43]; Taghipour et al. (2023) identified anticancer peptides from camel whey hydrolysates active against breast cancer cells [46]; Liu et al. (2023) found that a whey peptide-based enteral diet reduced tumor growth in colon tumor-bearing mice [47].

In the studies reviewed, various types of whey protein have been reported to have potential anticancer effects on different types of cancer, with most studies focusing on colorectal and breast cancer [28,37,38,39,40]. However, the quality and strength of this evidence vary across studies. Clinical trials indicated that high-purity whey protein supplementation improved nutritional status and reduced chemotherapy-induced toxicity [11,12].

The studies reported that whey proteins have various effects, such as antiproliferative [35], apoptosis-inducing [38], tumor-suppressing [36], metastasis-inhibiting [23], and increasing drug efficacy [30]. These findings are summarized in Table 3, which provides a detailed overview of direct cancer cell effects. Each type of effect was associated with a specific mechanism and measure of effectiveness [37,38,40,42,45]. Various anticancer properties were reported in the reviewed studies, and no single type of effect, mechanism, or measure of efficacy was consistently observed across multiple studies [30,37,38,42].

The anticancer mechanisms of whey proteins extend beyond direct tumor cytotoxicity and include systemic pathways such as antioxidant activity, immune modulation, anti-inflammatory effects, and gut microbiota regulation (Table 4). Each type of effect is associated with a distinct mechanism and a corresponding measure of efficacy. The review found one study for each effect type, mechanism, and efficacy measure. The same study reported two effects: anti-inflammatory and modulation of gut microbiota [45].

The studies examined reported that whey protein exhibits anticancer effects through four different mechanisms of action: internal [28] and external pathway activation [45], regulation of the cell cycle [37], and induction of endoplasmic reticulum (ER) stress [52]. These mechanisms were found to be associated with specific biological outcomes by targeting distinct molecular pathways (Table 5). The studies in question focused on two different types of cancer: colorectal cancer and multiple cancers.

In the studies examined, whey protein was found to exhibit anticancer effects through three distinct mechanisms: supporting glutathione synthesis, scavenging reactive oxygen species (ROS) [26], and iron chelation [6]. Each mechanism is associated with specific biological effects by targeting different molecular targets (Table 6). These antioxidant mechanisms protect against oxidative stress and contribute to cellular homeostasis in hepatocellular and colorectal cancer models.

Additionally, it has been reported that the anticancer effects of whey protein are mediated through four distinct mechanisms: inhibition of the AKT/mTOR pathway [28], modulation of the NF-κB pathway [38], regulation of the MAPK signaling pathway [42], and modulation of SIRT3 [44]. These interconnected signaling pathways show considerable crosstalk; inhibition of PI3K/Akt may suppress NF-κB activation, while SIRT3 modulation can influence mitochondrial metabolism and apoptosis regulation. It has been noted that each mechanism affects different molecular targets, and these effects are associated with the specific biological outcomes observed (Table 7).

A significant limitation identified across the studies is the heterogeneity of WP formulations. Their biological effects depend strongly on species sources (bovine, goat, camel), processing techniques (ultrafiltration, ion exchange, enzymatic hydrolysis), and degree of hydrolysis, which determine peptide composition and bioactivity. For example, trypsin-derived hydrolysates yield shorter cysteine-rich peptides with strong radical-scavenging potential, whereas alcalase or pepsin hydrolysates produce longer peptides that modulate apoptotic signaling [40,43]. Several studies identified specific bioactive peptides, such as lactoferricin and α-lactalbumin-derived fragments, with known amino acid motifs responsible for inducing apoptosis or scavenging ROS [42]. These structure–activity relationships highlight the importance of standardizing hydrolysis protocols in future studies. Such methodological diversity complicates direct comparison across studies and highlights the need for standardized preparation protocols in future research.

In conclusion, although studies report on the potential therapeutic applications of whey proteins in cancer management, the majority of evidence comes from preclinical models and small-scale clinical trials. Tsai et al. (2000) reported that whey protein may enhance the efficacy of conventional cancer drugs [30]. Additionally, whey protein isolate has been shown to increase the cytotoxicity of baicalein in hepatoma cells. Mazzuca et al. (2019) reported that whey protein supplementation benefits cancer patients undergoing treatment [12]. It has also been reported that patients with colorectal cancer who received whey protein supplementation experienced improved nutritional status and reduced chemotherapy toxicity. Bumrungper et al. (2018) have reported that whey protein isolate supplementation improves glutathione levels and immune function in chemotherapy-treated cancer patients [11]. Xiao et al. (2023) have demonstrated that they investigated the potential of whey proteins as carriers for anticancer drugs, enhancing drug delivery to cancer cells by utilizing their binding properties [16]. It is important to note that larger, well-designed clinical studies are needed to confirm these therapeutic potentials and determine optimal dosage and administration protocols [12].

Animal studies conducted by Hakkak et al. (2001) and Ronis et al. (2015) further demonstrated that whey protein diets may reduce the incidence of chemically induced tumors in rats [24,41]. Mohammed et al. (2019) reported that the antioxidant properties of whey protein, particularly its ability to enhance glutathione synthesis, protect against DNA damage and oxidative carcinogenesis [26]. Moreover, studies by Khan & Selamoglu (2019) and Cacciola et al. (2023) emphasized that whey proteins can modulate immune responses and gut microbiota composition, indirectly contributing to cancer prevention [45].

## 4. Conclusions

Whey proteins (WPs), particularly their bioactive peptides and hydrolysates, have emerged as promising adjuncts in cancer prevention and supportive care. This review summarizes a growing body of experimental and predominantly preclinical research demonstrating the potential anticancer activities of WPs, mainly in models of colorectal, breast, and liver cancers. These effects appear to be mediated through several key mechanisms, including the induction of apoptosis, regulation of oxidative stress, suppression of tumor proliferation, modulation of the gut microbiota, and inhibition of oncogenic pathways such as the PI3K/Akt, Wnt/β-catenin, and mTOR pathways.

What distinguishes this review is its integrated perspective, which connects molecular mechanisms with cellular and systemic outcomes, providing a comprehensive picture of WP’s possible therapeutic relevance rather than established clinical efficacy. Unlike many conventional therapies that often come with significant toxicity, WP-based interventions offer a well-tolerated and accessible nutritional approach. Preclinical and limited clinical studies suggest that WP can enhance immune function and improve redox balance in colorectal cancer, boost chemotherapeutic sensitivity in breast cancer, and even support psychological well-being in animal models by modulating tryptophan metabolism and microbiota. Moreover, recent advances in nanotechnology have opened new possibilities for enhancing the bioavailability and tumor-targeting efficiency of WP-derived compounds.

From a methodological standpoint, the evidence presented in this review spans in vitro assays, animal models, and a limited number of clinical trials. In vitro studies consistently report dose- and time-dependent cytotoxicity of WP fractions against various cancer cells. In vivo studies confirm WP’s ability to delay tumor development and reduce tumor burden. Clinically, WP supplementation has been associated with improvements in nutritional status, immune responses, and quality of life, particularly in patients with cachexia or those undergoing chemotherapy.

Despite these encouraging findings, important limitations remain. Most of the current mechanistic understanding is derived from preclinical evidence, which may not fully capture the complexity of human cancer biology. Additionally, significant variability exists in the preparation and composition of WP products used across studies, including variations in peptide profiles, degree of hydrolysis, and concentrations of bioactive components. These inconsistencies highlight the need for standardized formulations and rigorous pharmacokinetic evaluation in future research.

To fully realize the clinical potential of WP, large-scale, well-designed randomized controlled trials are essential. Such studies should determine optimal dosing regimens, treatment durations, and patient populations. Furthermore, investigations into potential synergies between WP and standard oncologic therapies, as well as its role in precision nutrition, are warranted. The immunomodulatory and microbiome-mediated effects of WP also warrant further investigation, particularly in the context of personalized medicine.

In summary, whey proteins represent a versatile and biologically active nutritional strategy with promising—yet still preliminary—anticancer potential. Their favorable safety profile, accessibility, and multifaceted molecular actions position them as valuable candidates for inclusion in integrated cancer care. With further validation in well-controlled clinical trials, WP-based interventions may help bridge the gap between nutrition and oncology, offering a supportive avenue for enhancing patient outcomes in both preventive and therapeutic contexts.

This review has some limitations. First, the number of clinical trials was limited, with small sample sizes and heterogeneity in interventions. Second, variations in whey protein formulations across studies make cross-comparison difficult. Finally, most mechanistic insights originate from experimental models, and further translational research is required to establish clinical relevance.

## Figures and Tables

**Table 1:** Comparative Anticancer Mechanisms of Different Whey Protein Formulations.

Formulation	Main Mechanisms	Major Cancer Models
Whey Protein Concentrate (WPC)	Antioxidant activity, Glutathione synthesis, ROS scavenging	Colorectal, Breast
Whey Protein Isolate (WPI)	mTOR/PI3K inhibition, Immune activation	Liver, Breast
Whey Protein Hydrolysate (WPH)	Apoptosis induction, Caspase-3 activation, Cell cycle arrest	Colon, Mammary
Bioactive Peptides	Gut microbiota modulation, Anti-inflammatory effect	Prostate, Multiple myeloma

**Table 2:** Characteristics and Risk of Bias Assessment of Included Studies.

Study	Study Design	Cancer Type	Whey Protein Type	Key Outcomes	Risk of Bias Tool	Risk of Bias Judgment
De Simone et al., 2011 [37]	In vitro	Colon adenocarcinoma	Peptide fractions from buffalo cheese whey	Reduced cell viability, cell cycle arrest	Reproducibility criteria	High
Eason et al., 2006 [38]	Experimental animal	Mammary carcinoma	Whey protein hydrolysate	Decreased tumor incidence, delayed tumor appearance	ARRIVE checklist	Some concerns
Attaallah et al., 2012 [39]	Experimental animal	Colon cancer	Whey protein concentrate and hydrolysate	Reduced tumor development	ARRIVE checklist	Some concerns
Cacciola et al., 2022 [40]	Experimental animal	Colorectal cancer	Delactosed milk whey	Activation of necroptosis and apoptosis pathways	ARRIVE checklist	Some concerns
Badr et al., 2021 [28]	In vitro	Multiple myeloma	Camel whey protein	Reduced cell viability, induced apoptosis	Reproducibility criteria	Some concerns
Mohammed et al., 2019 [26]	Experimental animal	Hepatocarcinoma	Whey protein concentrate	Alleviated liver carcinoma markers, improved antioxidant defense	ARRIVE checklist	Low
Ronis et al., 2015 [24]	Experimental animal	Mammary tumors	Whey protein hydrolysate	Reduced tumor incidence and increased tumor latency	ARRIVE checklist	Low
I.Sabancılar & Durak, 2022 [23]	In vitro	Breast cancer	Whey protein (unspecified)	Reduced cell viability	Reproducibility criteria	High
Hakkak et al., 2001, [41]	Experimental animal	Colon cancer	Whey protein (unspecified)	Reduced tumor incidence	ARRIVE checklist	Some concerns
I.Sabancılar, 2022 [36]	In vitro	Colorectal adenocarcinoma	Sheep whey protein	Reduced cell viability	Reproducibility criteria	Low
Aksoy et al., 2023 [42]	In vitro	Breast cancer	Whey protein derivatives	Decreased cell viability and migration	Reproducibility criteria	Some concerns
Dreanca et al., [43]	Experimental animal	Colon carcinoma	Whey beverage and concentrate	Reduced tumor volume, increased glutathione levels	ARRIVE checklist	Low
D’onofrio et al., 2021 [44]	In vitro	Colorectal cancer	Whey extracts	Inhibited cell proliferation, induced apoptosis	Reproducibility criteria	Low
Tsai et al., 2000 [30]	In vitro	Hepatoma	Whey protein isolate	Enhanced cytotoxicity of anticancer drugs	Reproducibility criteria	Some concerns
Ali & Elsebaie, 2018 [29]	In vitro	Breast and lung cancer	Whey protein isolate	Cytotoxic activity against cancer cells	ARRIVE and reproducibility	Some concerns
Cacciola et al., 2023 [45]	Experimental animal and in vitro	Colorectal cancer	Deactylated buffalo milk whey	Reduced tumor incidence, induced apoptosis	ARRIVE checklist	Some concerns
Taghipour et al., 2023 [46]	In vitro	Breast cancer	Whey protein hydrolysates	Reduced cell viability	Reproducibility criteria	Low
Rosa et al., 2020 [27]	In vitro	Prostate cancer	Whey beverages	Antiproliferative and apoptotic effects	ARRIVE checklist	Low
Liu et al., 2023 [47]	Experimental animal	Colon tumor	Whey peptide-based enteral diet.	Reduced tumor weight, increased apoptosis	ARRIVE checklist	Some concerns
Murali et al., 2021 [35]	In vitro	Colon carcinoma	Camel whey protein hydrolysates	Inhibited cell growth, induced cell cycle arrest	Reproducibility criteria	Low
Duarte et al., 2011 [48]	In vitro	Breast cancer	Lactoferrin	Decreased cell viability, increased apoptosis	Reproducibility criteria	Low
Mazzuca et al., 2019 [12]	Randomized controlled trial	Colorectal cancer	Highly purified whey protein	Improved nutritional status, reduced chemotherapy toxicity	Cochrane RoB 2.0	Some concerns
Boukhettala et al., 2010 [49]	Experimental animal	chemotherapy-induced mucositis	Whey protein (unspecified)	Improved nutritional outcome, reduced intestinal mucositis.	ARRIVE checklist	Some concerns
Bumrungpert et al. [11]	Randomized controlled trial	Various cancers	Whey protein isolate	Improved nutritional status, increased glutathione levels	Reproducibility criteria	Low

**Table 3:** Direct Cancer Cell Effects.

Effect Type	Mechanism	Effectiveness	Study Reference
Antiproliferative	Cell cycle arrest	Significant reduction in cell viability	[37]
Apoptosis induction	Activation of apoptotic pathways	Increased apoptosis in cancer cells	[40]
Tumor suppression	Reduced tumor incidence and growth	Decreased tumor development in animal models	[50]
Metastasis inhibition	Decreased cell migration	Reduced cell migration in vitro	[42]
Enhanced drug efficacy	Synergistic effect with anticancer drugs	Improved cytotoxicity of chemotherapy	[30]

**Table 4:** Systemic and Indirect Anticancer Mechanisms.

Effect Type	Mechanism	Effectiveness	Study Reference
Antioxidant activity	Enhanced glutathione production	Improved antioxidant defense	[26]
Immune enhancement	Stimulation of the immune response	Potential boost in anticancer immunity	[51]
Anti-inflammatory	Modulation of inflammatory markers	Reduced inflammation in cancer models	[45]
Gut microbiota modulation	Alteration of microbial composition	Potential indirect anticancer effects	[45]
Metastasis inhibition	Decreased cell migration	Reduced cell migration in vitro	[42]
Enhanced drug efficacy	Synergistic effect with anticancer drugs	Improved cytotoxicity of chemotherapy	[30]

**Table 5:** Apoptosis Pathways.

Mechanism	Target Molecules	Observed Effects	Cancer Type
Intrinsic pathway activation	Cytochrome C, Bcl-2 family proteins	Increased apoptosis	Multiple myeloma
Extrinsic pathway activation	Caspase-3, PARP-1	Enhanced apoptotic signaling	Colorectal cancer
Cell cycle regulation	Cyclin A, p21, p53	Cell cycle arrest	Colon adenocarcinoma
Endoplasmic Reticulum (ER) stress induction	PERK/IRE1/XBP1, CHOP	Increased ER stress-mediated apoptosis	Colorectal cancer

**Table 6:** Antioxidant Properties.

Mechanism	Target Molecules	Observed Effects	Cancer Type
Glutathione synthesis	Glutathione (GSH), Glutathione S-transferase alpha (GST-α)	Enhanced antioxidant defense	Hepatocarcinoma
Reactive Oxygen Species (ROS) scavenging	Superoxide dismutase, Catalase	Reduced oxidative stress	Colorectal cancer
Iron chelation	Lactoferrin	Prevention of iron-induced oxidative damage	Various cancers

**Table 7:** Cell Signaling Modulation.

Mechanism	Target Molecules	Observed Effects	Cancer Type
Protein kinase B (AKT)/mammalian target of rapamycin (mTOR) pathway inhibition	AKT, mTOR	Reduced cell proliferation and survival	Multiple myeloma
NF-κB pathway modulation	NF-κB, IκB	Altered inflammatory response	Colorectal cancer
MAPK pathway regulation	ERK, JNK, p38	Modified cell growth and apoptosis signaling	Modified cell growth and apoptosis signaling
SIRT3 modulation	SIRT3, PPAR-γ, PPAR-α	SIRT3, PPAR-γ, PPAR-α	Colorectal cancer

## Data Availability

The data and materials that support the findings of this study are available from the corresponding author upon reasonable request.
